# Simulating Real-World Slips: Enhanced Kinematic and Neuromuscular Responses to Experimental Slips in the Early vs. Late Stance Phase in Young and Older Adults

**DOI:** 10.3390/bioengineering12121284

**Published:** 2025-11-21

**Authors:** Marina Geissmann, Nicole Sarah Holliger, Lennart Carlson Neumann, Antonia Maria Eilfort, Linard Filli

**Affiliations:** 1 Swiss Center for Movement Analysis (SCMA), Balgrist Campus AG, 8008 Zurich, Switzerland; marina.geissmann@balgristcampus.ch; 2 Neuroscience Center Zurich, University of Zurich, 8008 Zurich, Switzerland; nicole.holliger@balgrist.ch (N.S.H.); lennart.neumann@balgrist.ch (L.C.N.); antonia.eilfort@balgrist.ch (A.M.E.); 3 Department of Health Sciences and Technology, ETH Zurich, 8093 Zurich, Switzerland; 4 Spinal Cord Injury Center, Balgrist University Hospital, University of Zurich, 8008 Zurich, Switzerland

**Keywords:** slip responses, mechanical perturbations, automatic postural responses, reactive balance, fall prevention, fall simulation, reactive balance training, slipping

## Abstract

Slips are a leading cause of injury and hospitalization among at-risk individuals. Replicating real-world slips by experimental, mechanical perturbations is essential for characterizing reactive balance mechanisms activated during near-fall situations and for training these mechanisms in fall prevention programs. This study compared treadmill-based, slip-like perturbations targeting the early (early perturbations, EP) vs. late stance phase (late perturbations, LP) in 22 young and 21 older adults. Biomechanical and neuromuscular responses were assessed using full-body kinematics and surface electromyography (EMG). Additionally, participants provided subjective rating of perturbation intensity and inconvenience. EP elicited stronger reactive balance responses than LP, characterized by greater deviations in leg joint and trunk kinematics, as well as shorter EMG onset latencies and enhanced EMG peak amplitudes. Gait parameters required longer to recover to baseline following EP than LP. Subjectively, EP were rated as more intense and inconvenient, and were perceived to more closely mimic real-world slips. Older adults showed delayed and attenuated reactive balance responses compared to younger adults. These findings highlight the importance of targeting the vulnerable early stance phase to accurately simulate real-world slip events. Such perturbation paradigms may support the development of more effective, task-specific perturbation-based training programs aimed at reducing falls in at-risk populations.

## 1. Introduction

Falls are a major cause of injuries and hospitalization among older adults and individuals with musculoskeletal or neurological disorders [1,2]. More than one-third of older adults experience at least one fall per year [3]. A significant proportion of injury-related falls occur due to slips during walking that result in a sudden loss of balance [4].

Slips occur when the friction coefficient (available friction) is lower than the ratio of shear to normal foot forces (required friction). The initial stance phase, immediately after foot contact, is the most vulnerable period for slips in the real-world setting [5,6], causing the foot to slide forward. Previous studies have demonstrated that the timing of perturbation onset within the gait cycle significantly influences the magnitude of the reactive response [7,8]. Intriguingly, most treadmill-based slip paradigms trigger belt deceleration at foot contact, resulting in peak slip velocity occurring during mid-to-late stance [9,10,11,12]. In contrast, experimental slips peaking during the critical early stance phase remain rare, likely due to methodological challenges in initiating perturbations prior to foot contact.

Previous studies investigating the biomechanical aspects of slips have employed oily surfaces [13,14,15] and moveable platforms [8,16]. Whereas these methods effectively replicate the mechanical characteristics of slipping, they are limited in their ability to randomize the timing or location of the perturbation due to their fixed positioning. Consequently, participants often anticipate slip events (e.g., by adopting a more cautious gait), which substantially confounds the interpretation of stabilizing responses. In contrast, treadmill-based perturbations allow for fully unpredictable slip timing and enable the collection of a large number of steps and perturbation events. Treadmill-based systems also provide high reliability in delivering perturbations with consistent amplitude and precise timing within specific phases of the gait cycle [9,10,17]. Therefore, treadmill-based perturbations offer a valid and accurate approach for examining fast postural responses and locomotor recovery following slips. While the effects of perturbation intensity on slip-induced falls have been extensively investigated [18,19,20], the influence of perturbation timing on slipping remains poorly understood.

Locomotor adaptability typically declines with age, becoming more challenging and less effective [21]. This is largely attributed to age-related neuromuscular and sensory deterioration, decreased tendon stiffness, and reduced muscle strength, all of which impair motor control and balance [21,22]. However, the impact of aging on reactive balance remains inconclusive: while some studies report substantial differences between younger and older individuals [21,22,23], others show no significant age-related differences [12]. The most prominent age-related differences include attenuated EMG responses [22] and prolonged recovery times following perturbations in older adults [21,23].

This study aimed at developing a novel perturbation algorithm targeting the critical initial stance phase to more accurately replicate real-world slip conditions. Specifically, we compared the biomechanical effects of identical deceleration perturbations applied during either the early or late stance phase in younger and older adults using multimodal instrumented motion capture and EMG. We hypothesized that slip-like perturbations during early stance would induce stronger reactive responses and prolonged recovery phases than those applied during late stance. Furthermore, we expected older participants to exhibit more pronounced perturbation responses and delayed recovery than their younger counterparts.

The findings of this study advance our understanding of how perturbation timing and age influence reactive responses to slipping. By establishing an experimental paradigm that closely replicates key features of real-world slips, this work may inform the development of more effective, task-specific training protocols to improve reactive balance and reduce fall risk.

## 2. Materials and Methods

### 2.1. Participants

Twenty-two healthy young and 22 healthy older adults were included in this study. No *a priori* power analysis was conducted to determine the sample size. However, a population size of approximately 20 healthy participants is frequent in studies examining the effects of mechanical perturbations on biomechanical aspects [10]. Only participants able to walk at least 30 min without assistance or rest were included. Exclusion criteria included pregnancy, breastfeeding, and any neurological or orthopedic conditions potentially affecting walking function or balance. One participant from the older group discontinued the study due to a muscle injury sustained during testing. Therefore, 22 young and 21 older adults were included in the final data analysis.

### 2.2. Experimental Protocol

Gait perturbations were applied using the GRAIL system (Motek, Houten, The Netherlands) that consists of ten motion capture cameras (Vicon, Oxford, UK) operating at 200 Hz, a split-belt treadmill with two embedded force plates sampling at 1000 Hz, and a 180° immersive virtual screen. Throughout the experiment, participants walked within an immersive virtual environment depicting a New York City scene (Motek, Houten, The Netherlands). Handrails were removed and participants were secured by a safety harness to prevent falls. Full body kinematics were recorded using a modified version of the Plug-in Gait model containing 36 markers. Surface EMG was performed using a wireless system (Myon Aktos, Cometa Systems, Bareggio, Italy) to assess activity in the vastus medialis (VM), semitendinosus (SM), tibialis anterior (TA) and gastrocnemius medialis (GM) muscles with a sampling frequency of 1000 Hz. Electrodes were placed on muscles of the leg ipsilateral to the perturbation (VM, SM, TA, GM) and the corresponding contralateral muscles (c_VM, c_SM, c_TA, c_GM). Electrode placement was conducted according to surface electromyography for the non-invasive assessment of muscles (SENIAM) guidelines where applicable (http://seniam.org (accessed on 24 July 2024)). Skin impedance was optimized by shaving and carefully abrading (Nuprep gel, Waver and Company, Aurora, CO, USA) the skin overlying the muscles of interest. The skin was then cleaned with alcohol prior to electrode placement.

Prior to the perturbation trials, all participants filled out the Activities-Specific Balance Confidence (ABC) scale questionnaire to assess the self-perceived balance confidence in performing various activities of daily life [24]. Additionally, the functional gait assessment (FGA) was conducted to measure the participants’ dynamic stability and balance [25]. Then, participants were familiarized with treadmill walking at different speeds for six minutes [26]. Thereafter, participants completed three perturbation blocks, each consisting of ten perturbations. They walked on the treadmill at a fixed speed of 1.2 m/s, while perturbations were induced through either deceleration or acceleration of the treadmill belt applied at different timepoints within the gait cycle. A fixed walking speed of 1.2 m/s was selected based on previous findings reporting it as a comfortable pace for both young and older adults [12]. Maintaining a constant walking speed across age groups ensured comparability of perturbation intensity and responses across participants.

For data analysis, we focused exclusively on the two deceleration perturbation types that mimic forward movements of the foot during slips. Importantly, both perturbations were identical in terms of their deceleration profile. Specifically, they were applied while participants walked at a fixed walking speed of 1.2 m/s. A deceleration of 5 m/s^2^ was imposed for 0.33 s, resulting in a negative peak belt velocity of −0.45 m/s. There was an immediate re-acceleration of the treadmill belt at 5 m/s^2^ for 0.33 s to return to the baseline speed of 1.2 m/s. However, the two slipping perturbations were applied at different timepoints within the gait cycle: the first deceleration algorithm was triggered upon foot contact, as defined by Zeni at al. [27], resulting in peak perturbation amplitude occurring during mid-to-late stance phase. The second deceleration algorithm was initiated during the swing phase, inducing the peak perturbation amplitude shortly after foot contact. Perturbations in which this peak velocity occurred before 5% of the gait cycle were excluded from analysis. For each perturbation, ten trials were assessed, with their order presented in a pseudo-randomized sequence to avoid anticipatory responses. At the end of the experiment, each perturbation type was individually evaluated by participants using self-reported ratings for perceived inconvenience and intensity (each reaching from 0 to 10), and their perceived similarity to a real-life situation causing instability.

### 2.3. Data Analysis

All data were analyzed using Vicon Nexus (version 2.10.3 Vicon Nexus, Oxford, UK) and custom-written Matlab scripts (version 2024a, The Mathworks, Natick, MA, USA). The marker data was filtered using a Woltring mean squared error (MSE) filter [28] and EMG data with a second order bandpass filter (10–500 Hz) followed by signal rectification. Gait events were identified by the algorithm developed by Zeni et al. [27] and manually corrected if needed. The following spatiotemporal parameters were analyzed: step length, step width, step duration and stance phase duration. Kinematic parameters were extracted from Plug-in-Gait. For each parameter, twenty gait cycles were analyzed prior to each perturbation to establish a baseline. Moreover, the perturbation step (Pert), as well as the ten steps post perturbation (P1 to P10) were examined to assess balance recovery. Cross-steps between treadmill belts were excluded from the analysis. Baseline EMG activity was determined over ten gait cycles without perturbations. Reaction time was defined as the point at which EMG activity exceeded the baseline mean + 2 standard deviations. EMG amplitude was evaluated by calculating the maximal change in the EMG signal between regular and perturbed walking conditions.

### 2.4. Statistical Analyses

Statistical analysis was performed using RStudio (version 4.4.2). The significance level was set at α = 0.05 for all statistical tests. Normality of the data was investigated using the Shapiro–Wilk test [29]. Normally distributed data sets were analyzed using parametric statistical tests, while those with non-normal distribution were assessed using non-parametric tests. Demographic differences between the young and older age group were investigated using independent t-tests (body height and body weight) or non-parametric Mann–Whitney tests (age, FGA and ABC scale) [30]. Gender distribution was tested using a 2-tailed Fisher’s Exact Test.

Peak velocity within the gait cycle was assessed using a paired t-test for LP, and a paired Wilcoxon test for EP, as the latter data set was not normally distributed. Subjective ratings of perturbation intensity and inconvenience, treated as ordinal data, were assessed using a Wilcoxon’s sign rank test.

Linear mixed models (Imer function of the lme4 package) were used to assess EMG reaction times and amplitudes, with perturbation (EP, LP) and muscle (SM, VM, GM, TA, c_SM, c_VM, c_GM and c_TA) included as fixed factors, and subjects as a random effect. For assessing recovery of spatiotemporal gait parameters (step length, step width, step duration and stance phase duration) upon perturbations, models were fitted with perturbation (EP, LP) and step (base, P1-P10) as fixed factors, and subjects as a random effect. A type-I ANOVA was performed on the fitted model, followed by Tukey’s honest significance difference (HSD) test for pairwise comparison.

Furthermore, one-dimensional continuous time series data, such as joint angles, were analyzed using MATLAB 2024a (The Mathworks, Natick, MA, USA) with the Statistical Parametric Mapping 1D approach (SPM 1D, www.spm1d.org) that is based on random field theory [25]. Data normality was assessed using the spm1d build in function (spm1d.stats.normality.anova1rm). Depending on the outcome of the data normality tests, either the statistical parametric mapping (spm1d.stats1d.anova1rm) or the statistical non-parametric mapping (spm1d.stats1d.nonparam.anova1rm) was applied. Post hoc analyses were conducted using the spm1d build in function (spm1d.stats1d.anova1_posthhoc) to compare the perturbations algorithms to normal walking. The null hypothesis was rejected if the SPM {t} exceeded the critical threshold t^*^ at alpha = 0.05.

## 3. Results

Twenty-two healthy young (11 females, 28.3 ± 4.8 years) and 21 older participants (10 females, 66.1 ± 3.9 years) were included in the analysis (Table 1). No significant group differences were observed in gender distribution (*p* = 1.0), body height (*p* = 0.1841; d = −0.411) and weight (*p* = 0.6923; d = −0.122). However, older participants showed reduced gait stability, as reflected by lower scores in the FGA (young vs. old: 29.8 ± 0.4 pts vs. 29.0 ± 0.9 pts; *p* = 0.0007; r = 0.520) and reduced balance confidence measured by the ABC scale (young vs. old: 97.6 ± 2.5% vs. 91.4 ± 10.4%; *p* = 0.0143; r = 0.375).

The timing of perturbation peak velocity within the gait cycle differed significantly between perturbation types in both age groups. In young adults, early perturbations (EP) occurred at 15.3 ± 2.8% and late perturbations (LP) at 47.5 ± 3.7% of the gait cycle (Z = 4.1069; *p* < 0.0001; rs = 0.6635). Similarly, in older adults, EP occurred at 15.9 ± 3.4% and LP occurred at 48.3 ± 5.2% of the gait cycle (Z = 4.0145; *p* < 0.0001; rs = 0.1143).

### 3.1. Initial Response to Experimental Slips in Young Participants

#### 3.1.1. Kinematics

**Figure 1:**
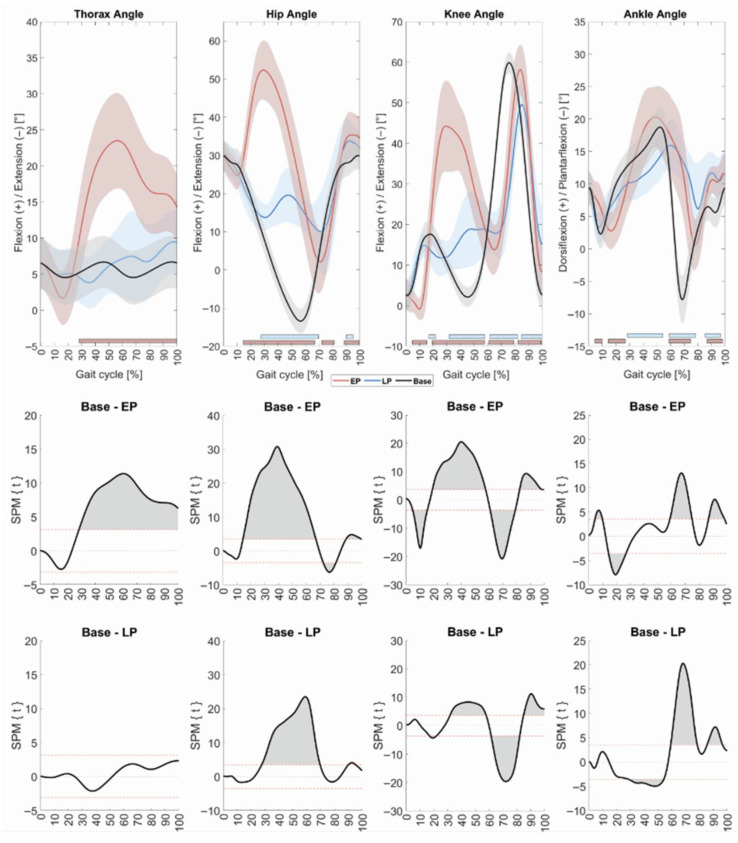
Initial kinematic responses to perturbations in young adults. (**Top row**) Mean sagittal plane kinematics of the thorax, hip, knee and ankle during regular walking (black lines; shaded areas represent ± 1 standard deviation), and in response to EP (red) and LP (blue). (**Middle row**) Statistical comparison between regular walking (base) and EP for thorax, hip, knee and ankle movements using Statistical Parametric Mapping (SPM). (**Bottom row**) SPM comparison between regular walking (base) and LP for all walking parameters. All data are time-normalized to the gait cycle. Joint angles for the hip, knee and ankle are shown for the leg ipsilateral to the perturbation. Significant differences between regular walking and EP are indicated by red bars at the lower part of the top figure row. Statistical differences between regular walking and LP are indicated by blue bars. Abbreviations: EP: early perturbations; LP: late perturbations.

Overall, EP elicited greater initial kinematic responses than LP (Figure 1). Specifically, EP induced enhanced trunk flexion relative to normal walking from 28.1 to 100% of the gait cycle (*p* < 0.0001). This marked abdominal flexion likely serves to shift the center of pressure (CoP) anteriorly, thereby counteracting backward destabilization and preventing falls. In contrast, LP did not result in altered trunk kinematics relative to normal walking.

Both EP and LP led to significant alterations of hip and knee angle trajectories, mainly manifesting as enhanced flexion during stance, and modified movement patterns during the swing phase. For EP, significant deviations were observed at the hip from 14.4 to 67.0% of the gait cycle (*p* < 0.0001) and at the knee from 4.8 to 15.4% (*p* = 0.0002), 19.1–57.7% (*p* < 0.0001), 61.0–79.2% (*p* < 0.0001). For LP, significant changes occurred at the hip from 27.6 to 69.8% of the gait cycle (*p* < 0.0001), and at the knee from 16.9 to 21.8% (*p* = 0.0073), 31.7–57.9% (*p* < 0.0001), 61.5–82.2% (*p* < 0.0001). Additional deviations from normal joint angle trajectories were observed during the swing phase, likely as a consequence of the pronounced initial flexion. For EP, significant deviations were noted at the hip between 72.2 and 81.4% (*p* = 0.0054) and 88.6–99.8% (*p* = 0.003), and at the knee between 82.3 and 99.0% (*p* < 0.0001) and 99.3–100% (*p* = 0.0166). For LP, deviations were present at the hip from 90.2 to 95.4% (*p* = 0.0113), and at the knee from 84.9 to 100% (*p* < 0.0001). Overall, aberrant flexion movements at both the hip and knee were substantially more pronounced following EP compared to LP (Figure 1).

Perturbation-induced deviations in ankle angle trajectories were generally comparable between EP and LP. EP elicited a delayed ankle plantarflexion after initial contact, with deviations emerging between 4.6 and 9.9% (*p* = 0.0068) and 14.5–27.0% (*p* = 0.0001)) of the gait cycle relative to normal walking. In contrast, LP induced a recued dorsiflexion during mid-stance, with significant alterations occurring between 28.5 and 54.2% (*p* < 0.0001) of the gait cycle. Later in the gait cycle, deviations were similar between EP and LP (EP vs. normal: 59.0–75.0% (*p* < 0.0001) and 87.0–98.5% (*p* = 0.0003): LP vs. normal: 59.2–78.4% (*p* < 0.0001) and 85.6–96.7% (*p* = 0.0009)).

#### 3.1.2. EMG

**Figure 2:**
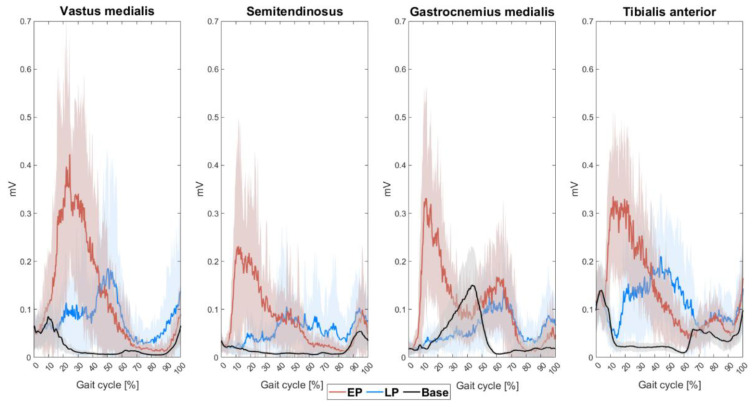
EMG responses to perturbations in various leg muscles of young adults. EMG activity of the vastus medialis, semitendinosus, gastrocnemius medialis and tibialis anterior muscles during regular walking (black curves), and in response to EP (red) and LP (blue). Shaded areas represent ±standard deviation for the respective conditions. All data are time-normalized to the gait cycle. Abbreviations: EP: early perturbations; LP: late perturbations.

Muscle activity profiles of the VM, SM, GM and TA on the perturbed side revealed substantial initial responses induced by both EP and LP (Figure 2). Across all muscles, EMG responses to EP appeared earlier within the gait cycle compared to LP, consistent with the earlier timing of EP application (Figure 2). Additionally, the magnitude of perturbation-induced EMG activity was greater in response to EP than LP.

EMG reaction times revealed significant main effects of *perturbation* (F(1,315) = 113.31, *p* < 0.0001), *muscle* (F(7,315) = 45.80, *p* < 0.0001), and their interaction (perturbation*muscle: F(7,315) = 67.17, *p* < 0.0001). Post hoc analysis demonstrated significantly shorter reaction times following EP vs. LP in the ipsilateral GM, as well as in the VM, TA and GM contralateral to the perturbation (Table 2). In contrast, reaction times in the ipsilateral VM were prolonged during EP, a phenomenon that might be attributed to the sudden passive extension of the ipsilateral hip and knee that potentially inhibits VM activity.

Maximal EMG amplitudes were significantly affected by the main factors *perturbation* (F(1,315) = 136.52, *p* < 0.0001) and *muscle* (F(7,315) = 17.84, *p* < 0.0001), as well as their interaction (F(7,315) = 9.85, *p* < 0.0001). Initial EMG responses showed enhanced amplitudes upon EP vs. LP across all muscles (Table 2). This effect reached statistical significance in all muscles, except the SM and GM on the contralateral side.

### 3.2. Recovery Response in Young Participants

A significant main effect of *perturbation* was observed in step length (F(1,441) = 4.64, *p* = 0.03174), step duration (F(1,441) = 21.57, *p* < 0.0001) and stance phase (F(1,441) = 31.61, *p* < 0.0001). Moreover, the main factor *step after perturbation* significantly affected step length (F(10,441) = 11.00, *p* < 0.0001), step width (F(10,441) = 9.58, *p* < 0.0001), step duration (F(10,441) = 133.10, *p* < 0.0001) and stance phase (F(10,441) = 149.13, *p* < 0.0001). A significant interaction effect of step after perturbation*perturbation was observed in step length (F(10,441) = 3.07, *p* = 0.0009), step duration (F(10,441) = 10.29, *p* < 0.0001) and stance phase (F(10,441) = 8.66, *p* < 0.0001).

**Figure 3:**
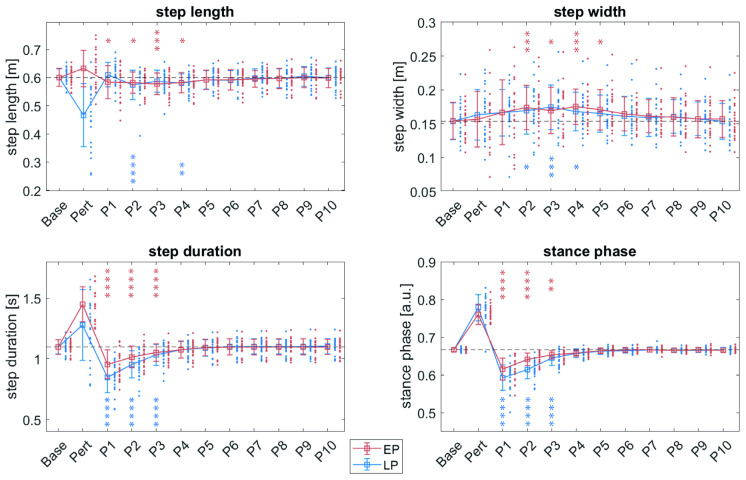
Recovery of spatiotemporal gait parameters following EP (red) and LP (blue) in young participants. The baseline (Base; dashed horizontal line) represents average values during regular walking, calculated from 20 unperturbed steps preceding the perturbation. Pert denotes the actually perturbed step, and P1-P10 indicate the ten subsequent recovery steps. Stance phase is reported as proportion of stance phase relative to step duration. Asterisks denote statistical significance: * *p* < 0.05, ** *p*-value < 0.01, *** *p*-value < 0.001, and **** *p* < 0.0001. Red asterisks indicate significant differences between EP and baseline; blue asterisks highlight differences between LP and baseline.

The time required for gait parameter recovery to baseline levels was slightly prolonged following EP compared to LP (Figure 3). After EP, step length returned to baseline by step P5, step width by P6, step duration and stance phase by P4. In contrast, following LP, step length was not significantly different from normal walking for two consecutive steps, and therefore no clear recovery phase was identified for this parameter. Step width recovered by P5, step duration and percentage of stance phase by P4 (Figure 3).

### 3.3. Subjective Perception of Perturbations in Young Participants

**Figure 4:**
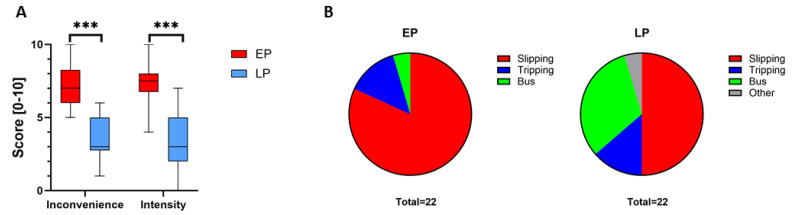
Subjective perception of the different perturbations in young adults. (**A**) Subjective ratings of EP (red) and LP (blue) with respect to perceived inconvenience and intensity. Rating ranged from 0 to 10, with 10 representing maximal inconvenience or intensity. (**B**) Comparison of EP and LP to real-life conditions including slipping, tripping, perturbations in bus and other conditions. *** represents *p*-value < 0.001.

Participants rated the degree of perturbation inconvenience significantly higher for EP (median = 7.0, 95% CI, 6.0, 8.0) than LP (median = 3.0, 95% CI, 3.0, 5.0, Z = −3.9859; *p* = 0.0002; rs = 0.0831; Figure 4). Similarly, perceived perturbation intensity was greater for EP (median = 7.5, 95% CI, 7.0, 8.0) compared to LP (median = 3.0, 95% CI, 2.0, 5.0, Z = −3.9771; *p* = 0.0002; rs = 0.2652).

When asked how they perceived the perturbations, young participants most commonly associated EP with slipping (81.8%), followed by tripping (13.6%) and a sudden bus stop (4.6%; Figure 4). LP was mostly perceived as slipping (50%) followed by a sudden bus stop (31.8%), tripping (13.6%) and other daily activities (4.6%). Interestingly, slipping was more frequently associated with EP than LP (81.8% vs. 50%), indicating that treadmill belt deceleration during early stance more closely mimics real-life slipping than perturbations applied during late stance.

### 3.4. Age Differences

#### 3.4.1. Initial Response

Overall, kinematic responses to perturbations were largely similar between younger and older participants. Younger adults exhibited higher peak hip and knee flexion angles compared to older participants during both perturbations. In LP trials, significant knee angle differences were observed throughout both the stance and swing phase in the younger group. In contrast, older participants only showed significant alterations during the swing phase 61.6–84.0% (*p* < 0.0001) and 88.8–100% (*p* = 0.0038) (Figure S1).

A significant main effect of *age* was found for EMG reaction times (F(1,41) = 22.56, *p* < 0.0001). Additionally, significant *age* and perturbation*age effects were identified for the maximal amplitudes of initial EMG responses (F(1,41) = 4.56, *p* = 0.0388, F(1,615) = 21.88, *p* < 0.0001). Older adults exhibited prolonged reaction times and reduced EMG amplitudes compared to younger participants.

Both age groups demonstrated shorter EMG reaction times for EP vs. LP in the same set of muscles (ipsilateral VM and, contralateral VM, TA and GM). However, the increase in maximal amplitudes of EMG responses was attenuated in older participants (Table 3). In this group, enhanced maximal amplitudes were only observed in the SM (*p* = 0.0195), VM (*p* < 0.0001), GM (*p* < 0.0001) and c_VM (*p* = 0.0012; Table 3).

#### 3.4.2. Recovery Response

In both age groups, comparable main effects of perturbation were observed for step length, step width, step duration and stance phase. However, subtle age-related differences emerged in the recovery time of spatiotemporal gait parameters. In older participants, step length recovered to normal walking at P4 following LP, whereas no significant deviation from normal walking was observed following EP, indicating no recovery steps were required (Figure S2). Step width recovered at P6 following EP and at P5 following LP, consistent with the recovery pattern observed in young participants. For step duration and stance phase duration, older adults required one additional step to return to baseline values compared to younger participants. Specifically, recovery occurred at step P5 in the older group versus P4 in the younger group for both perturbation types (Figure 3 and Figure S2).

#### 3.4.3. Subjective Perception of Perturbations

Analogous to their younger counterparts, older adults rated perturbation inconvenience higher for EP (median = 5.0, 95% CI, 3.0, 6.0) compared to LP (median = 3.0, 95% CI, 2.0, 3.0; Z = −3.6031; *p* < 0.0001; rs = 0.5025; Figure S3). Similarly, perceived perturbation intensity was significantly greater for EP (median = 5.0, 95% CI, 4.0, 7.0) than LP (median = 3.0, 95% CI, 2.0, 3.0; Z = −3.8233; *p* < 0.0001; rs = 0.1667). Overall, subjective ratings of both inconvenience and intensity were lower in older than younger participants (Figure 4 and Figure S3).

In terms of perceived perturbation type, the majority of older participants associated EP with slipping (80.9%), followed by tripping (9.5%), a sudden bus stop (4.8%) and other daily activities (4.8%). For LP, slipping was also the most commonly mentioned perception (42.8%), followed by a sudden bus stop (28.6%) and tripping (28.6%; Figure S3).

## 4. Discussion

Slips are a leading cause of injury among older adults and individuals with musculoskeletal or neurological disorders. Therefore, the development of experimental perturbation paradigms that closely mimic real-life slips is essential to better understand near-fall biomechanics and to exercise the reactive balance capacities critical for fall prevention. In this study, we applied treadmill-based, slip-like perturbations at different timepoints during the stance phase to examine differences in (1) kinematic gait patterns, (2) neuromuscular responses, and (3) subjective perception between perturbation types. Our findings highlight distinct, multimodal differences between perturbations with identical deceleration profiles applied during early stance (EP) vs. late stance (i.e., LP). Notably, initial responses to EP were more pronounced in both full-body kinematics and neuromuscular activity, reflecting enhanced reactive balance responses to perturbations targeting early stance. Specifically, EP elicited EMG responses with shorter onset latencies and higher peak amplitude compared to LP. Additionally, recovery of gait parameters to baseline levels was prolonged following EP, although this difference was marginal. Subjectively, EPs were more frequently perceived as real-time slips and EPs were rated as more intense and inconvenient than LPs. Overall, these findings indicate that EP elicits stronger reactive balance responses, and that precise perturbation timing is key for accurately replicating real-world slips.

Age-related differences were generally moderate. The most prominent differences were related to the initial EMG responses that revealed prolonged onset latencies and reduced peak amplitudes in older adults compared to younger ones across both perturbation types. Additionally, the phasic modulation of rapid muscle responses to EP compared to LP was attenuated in older participants. These findings indicate that reactive balance is diminished with aging, potentially contributing to the reduced ability of older individuals to respond and recover from unexpected perturbations in real-world scenarios.

### 4.1. Initial Responses to Perturbations

Immediate kinematic and neuromuscular responses were generally more pronounced following EP than LP. The initial biomechanical reactions to EP align with previously reported kinematic patterns in response to slip-like perturbations [31]: thorax and knee angles were initially extended for a short period, likely reflecting passive motion induced by the forward acceleration of the treadmill belt. Simultaneously, hip angles exhibited a pronounced flexion. These immediate biomechanical adaptations may reflect either passive, slip-induced mechanics or automated postural responses. Later during stance, both the thorax and knee transitioned into marked flexion, likely to stabilize the body and to reposition the center of mass (CoM) within the base of support [32,33]. Notably, adequate knee flexion has been identified as a critical factor in fall prevention [34]. Although similar patterns were observed in response to LP, the initial biomechanical adaptations were significantly attenuated, suggesting reduced balance demands during LP trials.

EMG reaction times and amplitudes in young participants were in line with previous findings [8,34]. Overall, EMG reaction times were substantially shorter following EP compared to LP. A single exception to this pattern was observed in the ipsilateral VM, which may have been initially suppressed due to the marked, passive hip flexion and knee extension induced by the perturbation. The more pronounced EMG responses following EP vs. LP align with a previous study applying slip-like over-ground perturbations [8], reinforcing the idea that the early stance phase—particularly initial foot contact—is a critical window for reactive balance responses. The phasic modulation of neuromuscular responses observed here is supported by observations that the central nervous system is dynamically modulated throughout the gait cycle. Notably, initial foot contact represents a particularly critical phase that is tightly controlled by descending motor systems and sensory afferents [35,36,37]. Enhanced neuromuscular control during initial foot contact likely facilitates rapid, reflexive responses essential for fall prevention and aligns with the assumption that reactive balance is a key mechanism to recover from near-fall situations [34,38].

Our findings of enhanced kinematic and neuromuscular responses to EP vs. LP indicate that experimental slips targeting the early stance phase induce a stronger gait distortion, underscoring the importance of precisely timed perturbations to accurately replicate the biomechanics of near-fall slips commonly encountered in daily life. Optimized slip simulations can enhance the effectiveness of perturbation-based training (PBT), providing a more task-specific stimulus to engage the reactive balance mechanisms critical for fall prevention. This, in turn, may improve the real-world transferability of PBT outcomes [39]. Interestingly, previous findings indicate that both young and older adults are able to improve their reactive balance performance through repeated exposure to unexpected gait perturbations [9,40,41], highlighting the adaptability of the neuromuscular system and the potential of PBT as a practical intervention for reducing fall risk.

### 4.2. Recovery from Perturbations

Beyond the immediate biomechanical and neuromuscular responses, both perturbation types also elicited longer-lasting responses. Specifically, step length, step duration and stance phase duration were reduced, while step width increased. Perturbation-induced reduction in step length aligns with previous studies [12,42] and is commonly interpreted as a shift towards a more cautious gait pattern [43]. Similarly, the increase in step width upon slip-like perturbations was reported previously [12,44] and represents a key compensatory strategy to enhance lateral stability during walking [43,44]. The observed stance phase shortening was previously identified as a critical component of recovery following slips [45].

Overall, recovery of gait parameters to baseline performance was slower following EP than LP, although these differences were modest. Importantly, all gait parameters normalized within the first five steps post perturbation, consistent with previous findings [12].

### 4.3. Subjective Perception

Participants rated early perturbations (EP) as significantly more intense and inconvenient than late perturbations (LP), suggesting that EP was perceived as a more disruptive and demanding gait disturbance. These subjective assessments align closely with the objective biomechanical data, reinforcing the conclusion that slip-like perturbations targeting the initial stance phase elicit stronger reactive balance responses and more accurately simulate near-fall situation in the real-world. Additionally, EP was associated more frequently with the sensation of slipping, further supporting its ecological validity in replicating authentic slip events. Collectively, these subjective evaluations strengthen the quantitative findings and underscore the relevance of early stance perturbations for realistic slip simulation and fall prevention research.

### 4.4. Age-Related Differences in Responses to Perturbations

Previous research on age-related differences in kinematic and neuromuscular responses to perturbations has yielded inconsistent results. Whereas some studies showed age-related variations in perturbation responses [21,46], others found no significant differences [12]. In our study, the most prominent age-related differences emerged in the initial neuromuscular responses. Specifically, older adults revealed significantly delayed EMG response onset latencies and markedly reduced response amplitudes for both perturbation types compared to younger adults. These findings align with previous results reporting diminished EMG amplitudes following tripping perturbations in older individuals [22]. The latency of neuromuscular responses to unexpected perturbations has been demonstrated to be a key factor whether a participant is able to recover or falls [34,38]. Additionally, our results revealed that the phasic modulation of EMG responses—i.e., the ability to scale responses based on the timing of the perturbation—is markedly attenuated in older adults, corroborating earlier findings [8]. Unlike younger participants, older adults showed a limited ability to enhance EMG amplitudes in response to early perturbations (EP), suggesting a reduced adaptability of the neuromuscular system. Summarized, these findings suggest that reactive balance capacity is diminished in older adults, potentially impairing their ability to recover from unexpected perturbations in real-world scenarios [8,39]. This impairment might result from age-related declines in supraspinal motor drive, reduced myelination of nerve fibers and decreased motoneuron excitability [47,48,49,50]. In addition, reduced sensitivity of muscle receptors and age-related changes in muscle fiber composition may further compromise balance responses [51,52]. Our findings suggest that reflexive muscle responses are a sensitive and valuable marker of reactive balance capacity. Incorporating such measures into future fall prevention research may enhance the identification of at-risk individuals and inform targeted intervention strategies.

Age-related differences in initial kinematic responses were moderate. However, younger participants showed higher peak flexion angles at the hip and knee, likely reflecting stronger reactive balance strategies aimed at repositioning the CoM to regain stability. Differences in recovery response between young and old participants were generally small and inconsistent. Whereas step duration and stance phase recovered slightly slower in the older age group, no age-related difference was observed in step width. Notably, only younger participants showed a reduction in step length following early perturbations (EP), indicating a more adaptive response to maintain stability.

The overall moderate age-related differences in perturbation responses may be attributed to the relatively high physical fitness and functional capacity of the older participants in our study, which likely enables them to tolerate the perturbations well. Nonetheless, the observed attenuation of neuromuscular responses in this group suggests the presence of impairments in reactive balance, even within a healthy aging population.

### 4.5. Limitations

This study has several limitations. First, perturbations were applied during treadmill walking, and the treadmill continued running after each perturbation. In contrast, real-life slips often result in an immediate cessation of walking. However, a treadmill-based setup was chosen for its advantages in triggering repetitive perturbations with high temporal accuracy, consistent amplitude and clearly defined timepoints that cannot be anticipated by participants. Second, we employed a fixed walking speed of 1.2 m/s for all participants. While previous studies identified this speed as close to comfortable walking speed for both young and older individuals [12], it may have been more demanding for some older participants. However, a fixed walking speed facilitated the comparability of perturbation intensity and responses across participants, thereby strengthening the consistency and interpretability of our findings. Third, the present study focused on the effects of perturbation timing on gait pattern changes. The influence of different perturbation intensity (i.e., acceleration and duration of perturbation) has not been explored here, although this factor is known to affect the response to perturbations [18,19,20].

## 5. Conclusions

Our findings underscore the critical role of perturbation timing in shaping reactive responses in both younger and older adults. Slip-like perturbations targeting the vulnerable, early stance phase elicited substantially greater responses compared to identical perturbations addressing the late stance phase. This was evident in amplified reactive balance responses and prolonged recovery phases to regain baseline gait patterns upon EP. Notably, EP were also subjectively rated as more intense and inconvenient, and were perceived to more closely replicate real-word slip events than LP.

Age-related differences were primarily observed in the initial neuromuscular responses, with older adults exhibiting prolonged muscle onset latencies, decreased response amplitudes and diminished modulation across perturbation types. These deficits reflect impaired reactive balance control, which may contribute to the elevated fall risk in older adults.

Our findings highlight that experimental, slip-like perturbations targeting the early stance phase provide a more ecologically valid simulation of real-world slip conditions. As such, they represent a valuable tool for task-specific, perturbation-based balance training interventions aimed at fall prevention in individuals at increased risk.

## Figures and Tables

**Table 1:** Demographic characteristics of the young (*n* = 22) and older (*n* = 21) participant group. Values are presented as mean ± standard deviation. The Functional Gait Assessment (FGA) has a maximum score of 30 points, whereas the Activities-specific Balance Confidence (ABC) scale has a maximum score of 100%. Abbreviations: F: female; M: male.

	Young	Old	*p*-Value
Gender [M/F]	11/11	11/10	1.0
Age [years]	28.3 ± 4.8	66.1 ± 3.9	<0.0001
Height [m]	1.73 ± 0.08	1.70 ± 0.07	0.1841
Weight [kg]	69.3 ± 13.1	67.8 ± 11.0	0.6923
FGA [score]	29.8 ± 0.4	29.0 ± 0.9	0.0007
ABC scale [%]	97.6 ± 2.5	91.4 ± 10.4	0.0143

**Table 2:** Reaction times and peak amplitudes of initial EMG responses to EP and LP in various leg muscles of young participants. Values present the mean ± standard deviation. Statistics include effects sizes measures. (estimate (β) and t ratio (t)). Abbreviations: c_: contralateral; EP: early perturbations; GM: gastrocnemius medialis; LP: later perturbations; SM: semitendinosus; TA: tibialis anterior; VM: vastus medialis.

**Muscle**	**Reaction Time [ms]**				
	**EP**	**LP**	***p*-Value**	**Β**	**t**
SM	88 ± 14	104 ± 33	0.2420	15.73	1.172
VM	166 ± 40	120 ± 18	0.0035	−39.45	−2.941
TA	101 ± 12	105 ± 18	0.9703	−0.50	−0.037
GM	100 ± 26	383 ± 127	<0.0001	315.09	23.485
c_SM	90 ± 11	105 ± 25	0.5050	8.95	0.667
c_VM	77 ± 13	103 ± 34	0.0362	28.23	2.104
c_TA	92 ± 13	121 ± 28	0.0374	28.05	2.090
c_GM	112 ± 14	157 ± 81	0.0004	47.86	3.567
**Muscle**	**Amplitude [mV]**				
	**EP**	**LP**	***p*** **-Value**	**Β**	**t**
SM	0.37 ± 0.26	0.20 ± 0.16	0.0004	−0.1774	−3.558
VM	0.71 ± 0.25	0.35 ± 0.27	<0.0001	−0.3582	−7.182
TA	0.52 ± 0.15	0.39 ± 0.14	0.0078	−0.1336	−2.678
GM	0.58 ± 0.22	0.26 ± 0.10	<0.0001	−0.3253	−6.522
c_SM	0.36 ± 0.19	0.32 ± 0.19	0.5151	−0.0325	−0.652
c_VM	0.83 ± 0.39	0.38 ± 0.25	<0.0001	−0.4554	−9.131
c_TA	0.45 ± 0.14	0.31 ± 0.13	0.0073	−0.1347	−2.701
c_GM	0.37 ± 0.12	0.34 ± 0.13	0.5334	−0.0311	−0.624

**Table 3:** Reaction times and peak amplitudes (mean ± standard deviation) of initial EMG responses to EP and LP in various leg muscles of older participants. Statistics include effects sizes measures (estimate (β) and t ratio (t)). Abbreviations: c_: contralateral; EP: early perturbations; GM: gastrocnemius medialis; LP: later perturbations; SM: semitendinosus; TA: tibialis anterior; VM: vastus medialis.

**Muscle**	**Reaction Time [ms]**				
	**EP**	**LP**	***p*-Value**	**β**	**t**
SM	108 ± 12	123 ± 48	0.2892	14.6	1.062
VM	216 ± 60	151 ± 33	<0.0001	−64.6	−4.705
TA	110 ± 12	128 ± 21	0.2002	17.6	1.284
GM	99 ± 45	415 ± 63	<0.0001	315.9	23.016
c_SM	109 ± 24	127 ± 43	0.2088	17.3	1.260
c_VM	103 ± 28	135 ± 56	0.0227	31.4	2.290
c_TA	116 ± 20	179 ± 119	<0.0001	63.4	4.618
c_GM	138 ± 30	168 ± 50	0.0271	30.5	2.221
**Muscle**	**Amplitude [mV]**				
	**EP**	**LP**	***p*** **-Value**	**β**	**t**
SM	0.30 ± 0.09	0.20 ± 0.11	0.0195	−0.0940	−2.348
VM	0.47 ± 0.22	0.25 ± 0.16	<0.0001	−0.2273	−5.674
TA	0.47 ± 0.21	0.41 ± 0.15	0.0940	−0.0673	−1.680
GM	0.41 ± 0.16	0.22 ± 0.13	<0.0001	−0.1829	−4.566
c_SM	0.27 ± 0.08	0.30 ± 0.17	0.3495	0.0375	0.937
c_VM	0.46 ± 0.20	0.33 ± 0.17	0.0012	−0.1310	−3.271
c_TA	0.42 ± 0.16	0.36 ± 0.16	0.0940	−0.0673	−1.680
c_GM	0.34 ± 0.12	0.28 ± 0.12	0.1070	−0.0648	−1.617

## Data Availability

The datasets generated and analyzed during the current study are available from the corresponding author upon reasonable request. All data of this study is included in the manuscript.

## Supplementary Materials

The following supporting information can be downloaded at: https://www.mdpi.com/article/10.3390/bioengineering12121284/s1, The supplementary files (Figure S1, Figure S2 and Figure S3) have been uploaded separately. Figure S1. Initial kinematic responses to perturbations in older adults. Top row: Sagittal plane kinematics of the thorax, hip, knee and ankle during regular walking (black lines; shaded areas represent ±1 standard deviation), and in response to EP (red) and LP (blue). Middle row: Statistical comparison between regular walking (base) and EP for thorax, hip, knee and ankle movements using Statistical Parametric Mapping (SPM). Bottom row: SPM comparison between regular walking (base) and LP for all walking parameters. All data are time-normalized to the gait cycle. Joint angles for the hip, knee and ankle are shown for the leg ipsilateral to the perturbation. Significant differences between regular walking and EP are indicated by red bars at the lower part of the top figure row. Statistical differences between regular walking and LP are indicated by blue bars. Abbreviations: EP: early perturbations; LP: late perturbations. Figure S2. Recovery of spatiotemporal gait parameters following EP (red) and LP (blue) in older participants. The baseline (Base; dashed horizontal line) represents average values during regular walking, calculated from 20 unperturbed steps preceding the perturbation. Pert denotes the actually perturbed step, and P1-P10 indicate the ten subsequent recovery steps. Stance phase is reported as proportion of stance phase relative to step duration. Asterisks denote statistical significance: * *p* < 0.05, ** *p*-value < 0.01, *** *p*-value < 0.001, and **** *p* < 0.0001. Red asterisks indicate significant differences between EP and baseline; blue asterisks highlight differences between LP and baseline. Figure S3. Subjective perception of the different perturbations in older adults. (A) Subjective ratings of EP (red) and LP (blue) with respect to perceived inconvenience and intensity. Rating ranged from 0 to 10, with 10 representing maximal inconvenience or intensity. (B) Comparison of EP and LP to real-life conditions including slipping, tripping, perturbations in bus and other conditions. *** represents *p*-value < 0.001.
