# Supplementary material for: Simulating Real-World Slips: Enhanced Kinematic and Neuromuscular Responses to Experimental Slips in the Early vs. Late Stance Phase in Young and Older Adults
Source: Bioengineering (Basel). 2025 Nov 21;12(12):1284. doi: 10.3390/bioengineering12121284 (PMC12730134; doi:10.3390/bioengineering12121284)
Supplement: Supplementary file 1 [file bioengineering-12-01284-s001.zip › bioengineering-3981797-supplementary.pdf]

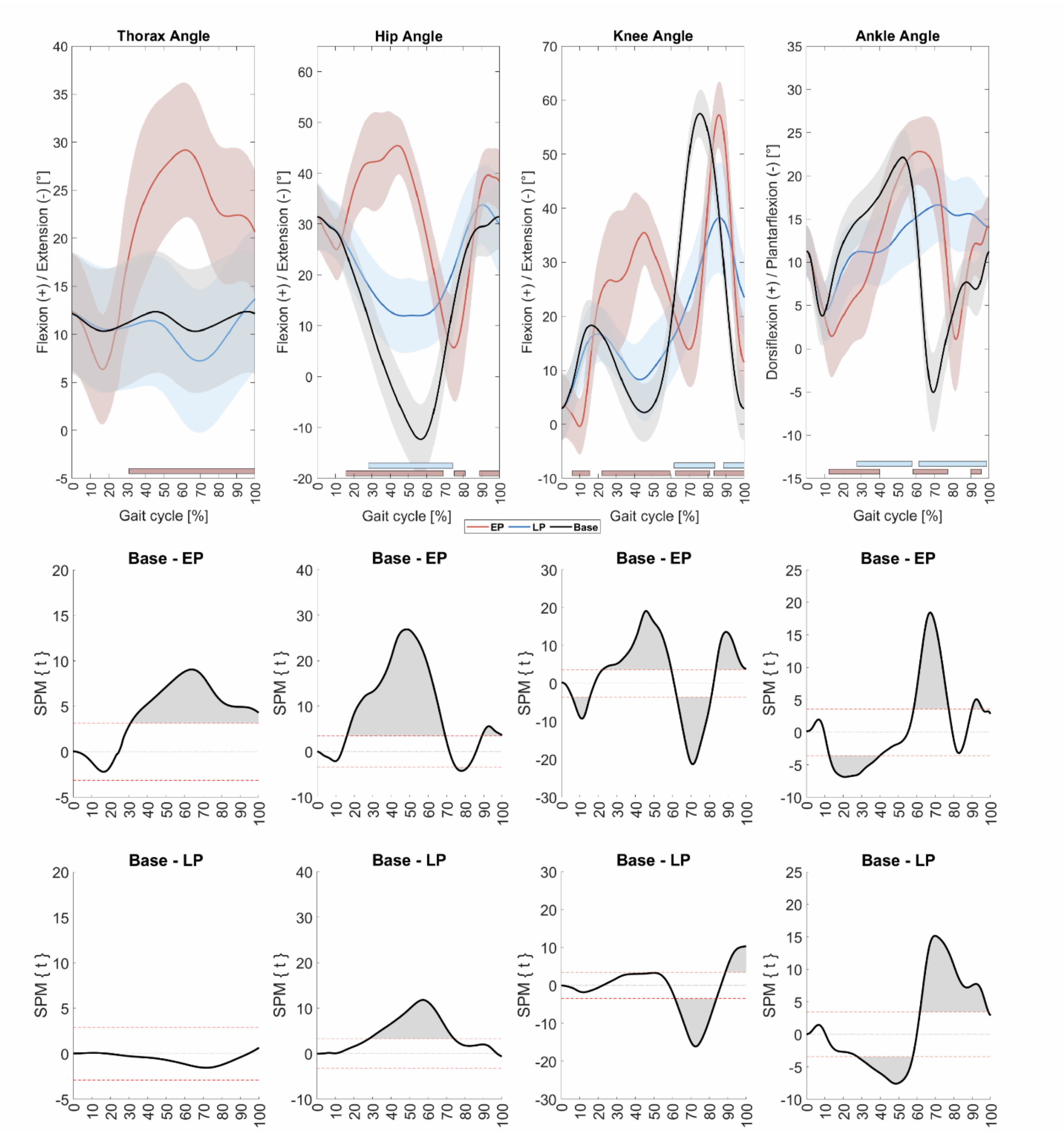

Figure S1. Initial kinematic responses to perturbations in older adults. **Top row:** Sagittal plane kinematics of the thorax, hip, knee and ankle during regular walking (black lines; shaded areas represent ± 1 standard deviation), and in response to EP (red) and LP (blue). **Middle row:** Statistical comparison between regular walking (base) and EP for thorax, hip, knee and ankle movements using Statistical Parametric Mapping (SPM). **Bottom row:** SPM comparison between regular walking (base) and LP for all walking parameters. All data are time-normalized to the gait cycle. Joint angles for the hip, knee and ankle are shown for the leg ipsilateral to the perturbation. Significant differences between regular walking and EP are indicated by red bars at the lower part of the top figure row. Statistical differences between regular walking and LP are indicated by blue bars. Abbreviations: EP: early perturbations; LP: late perturbations.

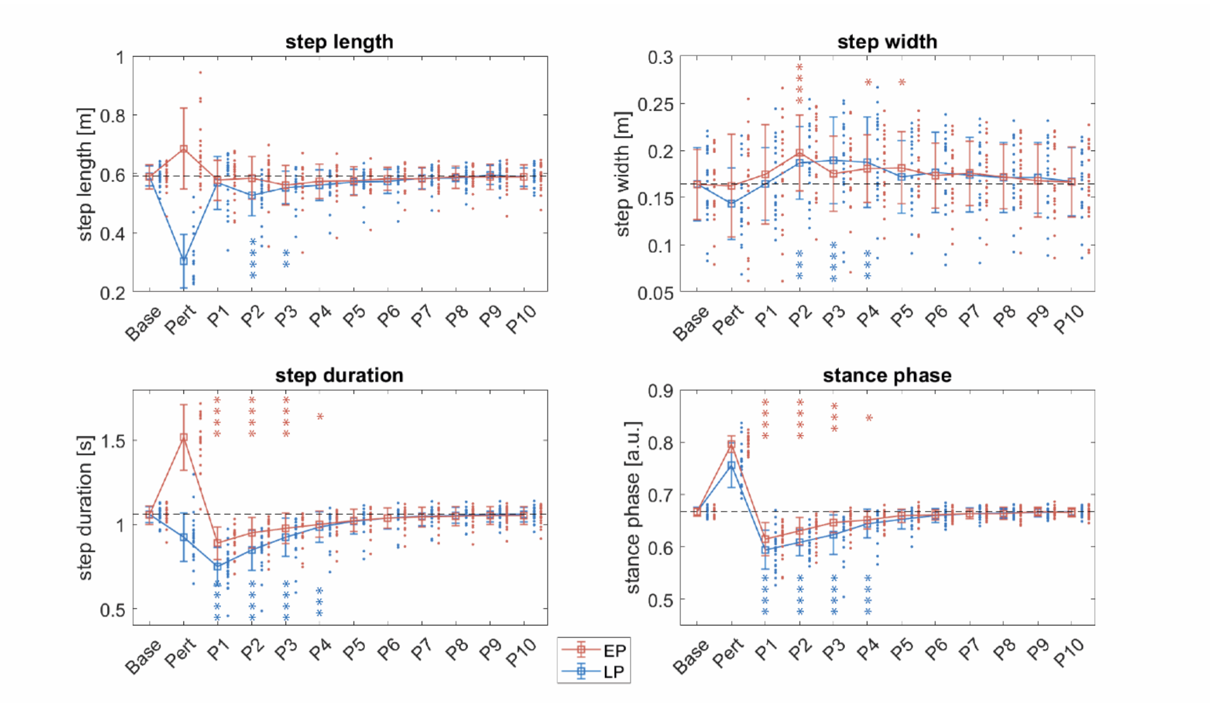

Figure S2. Recovery of spatiotemporal gait parameters following EP (red) and LP (blue) in older participants. The baseline (Base; dashed horizontal line) represents average values during regular walking, calculated from 20 unperturbed steps preceding the perturbation. Pert denotes the actually perturbed step, and P1-P10 indicate the ten subsequent recovery steps. Stance phase is reported as proportion of stance phase relative to step duration. Asterisks denote statistical significance: * p <0.05, ** p-value < 0.01, *** p-value < 0.001, and **** p < 0.0001. Red asterisks indicate significant differences between EP and baseline; blue asterisks highlight differences between LP and baseline.

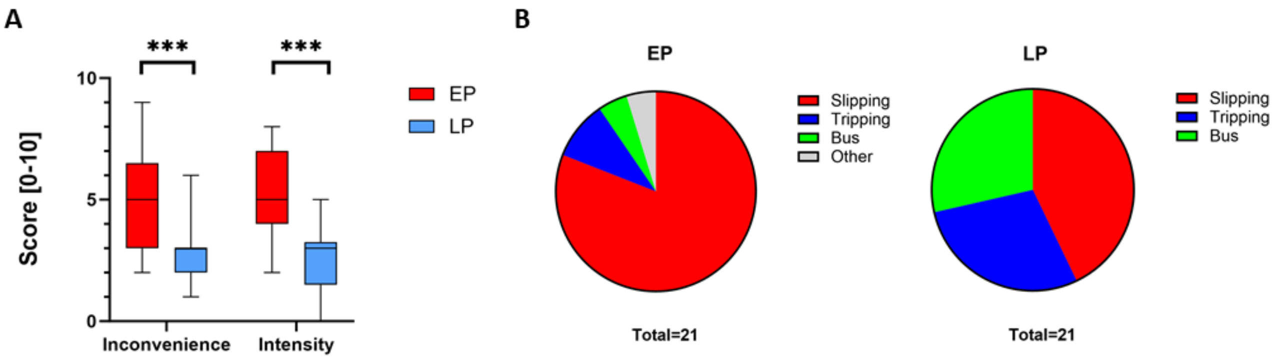

Figure S3. Subjective perception of the different perturbations in older adults. **(A)** Subjective ratings of EP (red) and LP (blue) with respect to perceived inconvenience and intensity. Rating ranged from 0 to 10, with 10 representing maximal inconvenience or intensity. **(B)** Comparison of EP and LP to real-life conditions including slipping, tripping, perturbations in bus and other conditions. *** represents p-value < 0.001.
